# Beverage Consumption Patterns among Norwegian Adults

**DOI:** 10.3390/nu8090561

**Published:** 2016-09-13

**Authors:** Mari Mohn Paulsen, Jannicke Borch Myhre, Lene Frost Andersen

**Affiliations:** Department of Nutrition, Institute of Basic Medical Sciences, University of Oslo, P.O. Box 1046 Blindern, Oslo 0317, Norway; j.b.myhre@medisin.uio.no (J.B.M.); l.f.andersen@medisin.uio.no (L.F.A.)

**Keywords:** beverage consumption pattern, meal types, food based dietary guide lines, alcohol consumption, sugar-sweetened beverages

## Abstract

Beverages may be important contributors for energy intake and dietary quality. The purpose of the study was to investigate how beverage consumption varies between different meals (breakfast, lunch, dinner, supper/evening meal, snacks) and between weekdays and weekend-days in Norwegian adults. A cross-sectional dietary survey was conducted among Norwegian adults (*n* = 1787) in 2010–2011. Two telephone-administered 24 h recalls were used for dietary data collection. Breakfast was the most important meal for milk and juice consumption, dinner for sugar-sweetened beverages and wine, and snacks for water, coffee, artificially sweetened beverages, and beer. Consumption of sugar-sweetened and artificially sweetened beverages did not differ between weekdays and weekend-days among consumers. The average intake of wine and beer (men only) was higher on weekend-days. Higher age was positively associated with wine consumption and negatively associated with consumption of water, sugar-sweetened, and artificially sweetened beverages. Higher education was associated with consumption of water, beer, and wine, whereas lower education was associated with sugar-sweetened beverage consumption. Beverage consumption patterns among Norwegian adults vary between different meal types and in subgroups of the population. Alcohol consumption was higher on weekend-days. Knowledge regarding beverage consumption patterns in the population should be considered when revising dietary guidelines in the future.

## 1. Introduction

Beverages may be important contributors for energy intake and overall dietary quality, in the same way as food [1,2,3]. We know, for instance, that milk is one of the most important contributors of calcium intake [4,5,6,7], while sugar-sweetened beverages contribute, in large part, to the intake of added sugar, especially among youths [6,8]. Sugar-sweetened beverage consumption has been associated with the worldwide obesity epidemic [9,10,11], although systematic reviews have highlighted the need for better randomized controlled trials, to investigate a causal effect [12,13]. High alcohol consumption has been associated with several acute and chronic conditions in a dose-response relationship [14]. Earlier studies have indicated a J-shaped association for alcohol consumption and all-cause mortality [15]. Recent studies have, in contrast, demonstrated that only women older than 65 years had lower mortality with low alcohol consumption [16]. A newly-published meta-analysis did not find any protective effects of a low-to-moderate alcohol consumption on all-cause mortality, compared to non-drinkers [17].

In the Norwegian food-based dietary guidelines (FBDGs), two of the twelve guidelines concern beverage intake; one regards avoidance of beverages and foods rich in added sugar on an everyday basis and one recommends drinking water when thirsty [18]. The same is found in international guidelines [19]. In Norway the consumption of sugar-sweetened beverages has been reduced from 63 to 55 liters per inhabitant from 2010 to 2014 [8]. The same trend is seen in Britain and the USA [20,21]. There are also specific advices concerning alcohol intake and the health authorities recommend reducing the consumption of alcohol-containing beverages [22]. Despite this, the consumption of alcoholic beverages in Norway has increased since the year 2000. The intake of wine per capita was at the highest level registered ever in 2013, while beer has had a slight reduction since the top levels in 2009 [8].

Despite dietary recommendations and knowledge regarding health effects of the consumption of different beverages, there is scarce knowledge concerning which meals and weekdays different beverages are consumed. There are few systematic analyses of overall beverage patterns and trends at the national and international level. It has been suggested that public health advice and strategies to change dietary intake need to focus on meal types to be understandable and usable by the population [23]. Kearney and coworkers describe that a consideration of eating patterns in the general population, including beverage consumption, is necessary when developing FBDGs [24]. Information regarding the distribution of food and beverage intake from different meals may provide important information for development and revision of FBDGs, understand habits, and for tailoring dietary interventions to the population [25]. 

Dietary behavior varies among different subgroups of the population and several studies have described an association between socio-economic position and dietary habits [26,27,28]. Less literature is published regarding beverage consumption habits and background variables in general, although the association with higher intakes of sugar-sweetened beverages in groups with lower socioeconomic status is well described [29,30,31,32]. Knowledge concerning how beverage consumption varies in relation to the background variables age, gender, education, smoking habits, and body mass index (BMI) may contribute to more specific and tailored beverage recommendations to the population. It may also be useful in understanding habits and designing dietary interventions. 

The aim of the present paper was to investigate the consumption of beverage types across meals, beverage intake during weekdays and weekend-days, and how beverage intake is associated with gender, age, education, smoking, and BMI.

## 2. Materials and Methods

### 2.1. Design and Participants

The present study was based on data from a Norwegian national dietary survey, Norkost 3, conducted in 2010–2011. The design and methodologies have earlier been described in detail [33]. A representative sample of the adult (18–70 years) Norwegian population (*n* = 5000) was randomly selected from the National register and asked to complete two 24 h recalls administered by telephone approximately four weeks apart. Data were collected about all days of the week in the study, but not for each individual. The distribution of interviews across the days of the weeks was the following: Mondays (21%), Tuesdays (18%), Wednesdays (18%), Thursdays (10%), Fridays (7%), Saturdays (7%), and Sundays (19%).

Of the 5000 persons invited, 153 were unavailable for contact. In total, 1787 participants completed two recalls (37% participation rate). Only participants completing both 24 h recalls were included in the analyses.

Every 25th participant was randomly selected to receive 3000.00 NOK. Feedback regarding the individual participant’s dietary composition was offered to those who wanted such feedback.

Verbal informed consent was collected from all participants. The study (2009/1318b) was approved by the Regional Committee for Medical and Health Research Ethics, 13 October 2009, and conducted according to the guidelines laid down in the Declaration of Helsinki.

### 2.2. Assessment of Beverage Intake

The 24 h recalls assessing food and beverage intake were performed by trained interviewers using a dietary assessment system (KBS version 7.0, University of Oslo/clave, Oslo, Norway) which is linked directly to a food composition database based on the Norwegian food composition table from 2006 [34]. Before starting the interview, the participants were asked if the previous day was considered a normal day with regard to food and beverage intake. Seventy-three percent of the recall days were considered as normal days by the participants. The respondents were encouraged to give detailed information regarding portion sizes of foods and beverages consumed. Amounts were quantified by household measures and aids in the form of a booklet including pictures of foods in different portion sizes and glasses/cups in different sizes. The booklet was sent by mail to all invited participants together with the invitation letter. The interviewers used a checklist of commonly forgotten food and drink items at the end of the interview to reduce the risk of underreporting.

### 2.3. Meal Types and Categorization of Beverage Types

Meal types were categorized as breakfast, lunch, dinner, supper, or snacks. Snacks also included only a beverage and intake of supplements. The most important meal for each beverage type was defined as the meal type with the highest average consumption in grams of the beverage type in question.

The beverage types included in the analyses were water, coffee, tea, milk, fruit juice, sugar-sweetened beverages, artificially sweetened beverages, beer, and wine. Water included both tap and bottled water. Sugar-sweetened beverages included soft drinks and squash with added sugar. Artificially sweetened beverages included soft drinks and squash with artificial sweeteners or without added sugar. The beer and wine included were all alcohol-containing.

### 2.4. Background Variables

Body mass index (BMI) was calculated based on self-reported weight (in kilograms) and height (in meters), as weight divided by the square of height (kg/m^2^). The continuous BMI variable was divided into two categories; “low and normal weight” (BMI < 25 kg/m^2^) and “overweight” (BMI ≥ 25 kg/m^2^). Level of education was reported into eight categories, but was merged to two categories: “high school, technical school, trade school or lower” and “university or college”. The continuous age variable was categorized into three age groups: 18–34 years, 35–54 years, and 55–70 years. Smoking habits were categorized into: “smokers” (daily/occasional smokers) and “non-smokers” (never-smokers and previous smokers). Interest in a healthy diet was reported into five categories ranging from “no interest” to “very high interest”, and this variable was categorized into: “no, low, or moderate interest” or “high or very high interest”.

### 2.5. Days of the Week

For the analyses regarding differences between weekdays and weekend days the days of the week were categorized as either weekday or weekend day. All meals during Monday to Thursday were categorized as weekday meals, whereas all meals during Saturdays and Sundays were categorized as weekend meals. It was assumed that dinner, supper, and snacks on Fridays were more like weekend meals and, therefore, categorized as weekend meals. While breakfast and lunch meals on Fridays were assumed to be more like weekday meals and, therefore, categorized as weekday meals.

### 2.6. Statistical Analyses

Statistical analyses were performed using Stata version 14.0 (StataCorp LP, College Station, TX, USA) and IBM SPSS Statistics 20.0 (IBM Corporation, Armonk, NY, USA). The analyses were performed separately for each type of beverage and all tests were two-sided. The data contained repeated measurements for each participant as the same participant could contribute with more than one meal to the analyses. To adjust for the dependency in the data due to repeated measurements for each participant, mixed models with total daily intake in grams of each beverage type as outcome variables were used with a variance component (random intercept) for participants. These adjustments were performed for the analyses of beverage consumption for different meals and differences in intake of selected beverage types between weekdays and weekend days. 

To identify the most important meal for each beverage type in each gender, meal type (breakfast, lunch, dinner, supper, and snacks) was added as an independent variable to the mixed model. Differences between genders in the most important meal were tested. In cases with significant differences between genders, men and women were analyzed separately. 

As the data contained a high number of zeros (meaning that the person had not consumed the beverage type in question for the respective meal or for the day in question), causing a violation of the assumption of normally distributed residuals, case bootstrapping with 1000 repetitions was performed for analyzes of average beverage consumption to different meals. The results are presented as adjusted means, bootstrap 95% confidence intervals, and bootstrap *p*-values. For some of the meal types the number of consumers of certain beverage types (e.g., beer for breakfast, tea for dinner) was zero or very small. In these specific cases, estimation of a 95% confidence interval was not possible.

In the analyses of differences between weekdays and weekend-days only consumers of the different beverage types were included. Adjustments were made for the categorical variables gender, BMI, normal day, smoking, interest in a healthy diet, education, and age.

To analyze the associations between background characteristics of the study participants and beverage consumption logistic regression was used (The participants were categorized as “users” or “non-users” of the different beverage types and these dichotomous variables were the dependent variables in the analyses. The models were adjusted for BMI, education, age, interest in a healthy diet, and smoking.

## 3. Results

### 3.1. Characteristics of the Study Population

Table 1 shows the background characteristics of the participants in the Norkost 3 survey. Fifty-two percent of the study participants were women and the mean age was 45 years for women and 47 years for men (age range 18–70 for both genders). In the Norkost 3 study, a higher percentage belonged to the highest age interval and a lower percentage to the youngest age interval, compared to the general population. The proportion with higher education was larger and the proportion of smokers was lower in Norkost 3 than in the general population [25].

### 3.2. Patterns of Beverage Consumption Related to Meals

Table 2 shows mean daily intake of beverages from each meal. The average intake to the most important meal was not different between genders for any of the beverage types, except for tea. Men had the highest intake of tea from breakfast, while women had the highest tea intake from snacks.

Milk and fruit juices were mainly consumed for breakfast. Dinner was the most important meal for sugar-sweetened beverages and wine, whereas snacks contributed to the highest intake of water, coffee, artificially sweetened beverages, and beer.

The average intake to the most important meal was not different between genders for any of the beverage types, except for tea. Men had the highest intake of tea from breakfast, while women had the highest tea intake from snacks.

Milk and fruit juices were mainly consumed for breakfast. Dinner was the most important meal for sugar-sweetened beverages and wine, whereas snacks contributed to the highest intake of water, coffee, artificially sweetened beverages, and beer.

### 3.3. Patterns of Beverage Consumption on Weekdays vs. Weekend Days

The proportion of participants consuming sugar-sweetened beverages, artificially sweetened beverages, beer, and wine on one or both recall days are presented in Table 3. On average, 34% of the participants were consumers of sugar-sweetened beverages, with the proportion being higher for men.

**Figure 1:**
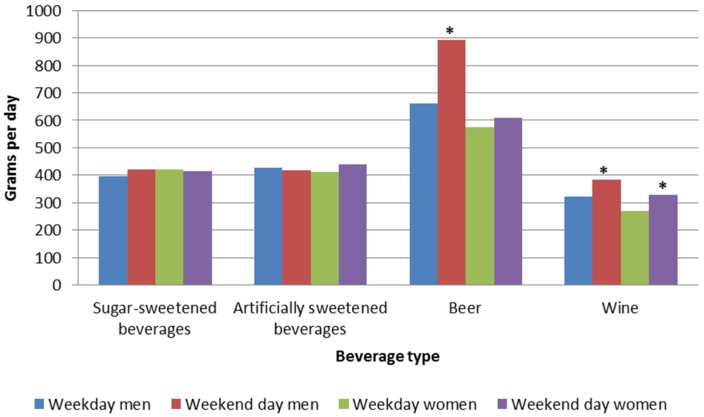
Average consumption in grams of sugar-sweetened beverages (*n* = 353 men and 260 women), artificially sweetened beverages (*n* = 191 men and 242 women), beer (*n* = 182 men and 85 women), and wine (*n* = 156 men and 204 women) among consumers of the selected beverage types on weekdays and weekend days. * *p* < 0.05 for difference between weekdays and weekend days. Tested with mixed models.

Figure 1 illustrates mean intakes of sugar-sweetened beverages, artificially sweetened beverages, beer and wine on weekdays and weekend-days for men and women (consumers only). 

The average consumption of both types of beverages among consumers was about 4 dL per day. The intakes of sugar-sweetened beverages and artificially sweetened beverages did not differ between weekdays and weekend days.

The average beer intake among consumers was 26% higher for men on weekend days than on weekdays, 891 g/day vs. 661 g/day, respectively (*p* = 0.028). For female beer consumers, no differences in beer intake were observed between weekdays and weekend days. Wine intake was higher on weekend days, compared to weekdays for both men (*p* = 0.044) and women (*p* = 0.016), the differences were rather modest; 16% (63 g) in men and 17% (57 g) in women.

### 3.4. Background Variables Associated with Intake of Different Types of Beverages

Table 4 shows how background characteristics were associated with users and non-users of different beverage types.

In general, water was consumed by a high proportion of participants (more than 90%) in all of the analyzed groups. Women were more frequent water drinkers with 92% higher odds of having consumed water on one or both recall days, compared to men. The oldest age group (55–70 years) had lower odds for consuming water than the younger participants (18–34 years) and there was a significant trend for less water consumption with increasing age. Additionally, those having a higher education and those reporting to have an interest for a healthy diet were more likely to be water consumers.

Milk consumption was not associated with any of the background variables analyzed, whereas juice intake was associated with being young, having a normal or low BMI, having a university or college education, and being a non-smoker.

Coffee intake showed a strong association with age. The oldest age group in the study population had almost five times higher odds of drinking coffee, compared to the youngest age group. Participants interested in a healthy diet also had higher odds of being a coffee consumer, compared to participants with no, low or moderate interest. Smokers had 70% higher odds of consuming coffee, compared to non-smokers.

Tea consumption was associated with all background variables analyzed. The factors associated with tea drinking were being a woman, being in the oldest age group, having a normal or low BMI, having a higher education, being interested in a healthy diet, and being a non-smoker.

For sugar-sweetened beverages, women were less likely to consume such beverages than men. The oldest age group had 76% lower odds of sugar-sweetened beverage consumption compared to the youngest participants. Participants with higher education and participants with high or very high interest in a healthy diet also had lower odds of being consumers of sugar-sweetened beverages.

In contrast to the gender differences observed for sugar-sweetened beverages, women had 38% higher odds of consuming artificially sweetened beverages compared to men. Participants in the oldest age group had 53% lower odds of drinking artificially sweetened beverages, while participants being overweight had higher odds of consuming artificially sweetened beverages, compared to participants with a normal or low BMI. People interested in a healthy diet had 21% lower odds of consuming artificially sweetened beverages, compared to people with no, low or moderate interest.

Women were less likely to have consumed beer than men. Having a university or college education was associated with 43% higher odds of drinking beer. Smokers were more likely to be beer consumers than non-smokers.

With regard to wine, participants in the oldest age group were almost four times more likely to be wine consumers compared to the youngest participants. Wine consumption was less prevalent among participants with a BMI ≥ 25 kg/m^2^ compared to those with a BMI < 25 kg/m^2^. Wine consumption was also positively associated with an interest of having a healthy diet, compared to having no, low, or moderate interest.

## 4. Discussion

The results showed that, in a Norwegian setting, breakfast was the most important meal for the intake of milk and juice. For tea, the main contributing meal differed between men and women, with breakfast being the most important meal for men and snacks being the most important meal for women. Dinner was the most important meal for sugar-sweetened beverages and wine, whereas snacks were the most important meal for water, coffee, artificially sweetened beverages, and beer. The intake of wine was higher on weekend days than on weekdays among consumers. The same accounts for beer intake among men. Higher age was found to have a strong association with consumption of coffee, tea, and wine, whereas younger age was associated with consuming water and sugar-sweetened beverages. Higher education was associated with consumption of water, juice, tea, and alcohol-containing beverages (beer and wine), whereas no education or education of lower degree was associated with consumption of sugar-sweetened beverages.

### 4.1. Patterns of Beverage Consumption Related to Meals

Milk and fruit juices, together with coffee and tea, have also been previously found to be the most commonly consumed beverages for breakfast in Norway and the Scandinavian countries. This is described in the survey “Eating patterns, a day in the life of Nordic people”, where computer assisted telephone interviews (CATI) were performed among 4800 Scandinavian individuals above 15 years of age [35].

Dinner was the most important meal for intake of sugar-sweetened beverages and wine. The average intake of sugar-sweetened beverages among all participants was ½ dL each day for dinner. One third (34%) of the participants were consumers of sugar-sweetened beverages. Among consumers the average daily intake was about 4 dL. The Norwegian health authorities recommend drinking water at meals and between meals because sugar-sweetened beverages increase the risk of obesity, tooth decay, and acid damage to teeth [22].

Snacks were the most important meal for water consumption in our study. A reason for this may be that water is regularly drunk between meals when thirsty and in association with physical activities. Snacks were also the most important meal for coffee, beer and artificially sweetened beverages. Coffee is a beverage that may be frequently consumed in social settings and at work. Beer may also be associated with social hang-outs in some groups of the population. Sieri et al. [36] found that alcohol was drunk outside main meals in most of the ten countries participating in the European Prospective Investigation into Cancer and Nutrition (EPIC) study. Italy was an exception to this, where most of the alcohol intake was consumed during meals [36]. Snacks were also found to be the most important meal for alcoholic drinks among 6000 participants in The Netherlands, Ireland, and the UK [24]. In a study of fluid intake in the French population most beverages were ingested during the main meals breakfast, lunch, and dinner, and only small amounts were consumed between meals [37]. This deviates somewhat from our results, where the highest average intake of several beverage types, particularly water and coffee, were consumed as part of snacks.

### 4.2. Patterns of Beverage Consumption on Weekdays Compared to Weekend Days

We observed that the average intakes of wine among wine-consumers (both men and women) and beer (men only) were higher during weekend days, compared to weekdays. This complies with results from a British study by Gibson and coworkers, where higher consumption of alcoholic drinks was observed during weekends, especially Saturdays [38]. Among almost 12,000 U.S. adults it was also found that energy intake from alcohol was higher on Saturdays, compared to weekdays [39]. For women, beer intake was not significantly higher on weekend days compared to weekdays in our study. Sieri et al. [36] found that alcohol consumption, particularly among women, increased markedly during the weekend in nearly all centers participating in the EPIC study. Exceptions from this were some centers in Germany and Spain for men and Italy for women. Among U.S. adults it was found, in contrast, that the weekend-weekday difference in energy intake from alcohol was larger among male adults than among women [39]. It seems like consumption habits with regard to wine and beer on weekdays compared to weekend days varies between different countries. Therefore, it may be reasonable to evaluate the situation in each country when giving public health advice. 

Surprisingly, we did not find any differences in the intake of sugar-sweetened and artificially sweetened beverages between weekdays and weekend days among consumers. There is scarce literature published regarding consumption of such beverages among adults. Among almost 800 Danish children and adolescents the intake of sugar-sweetened beverages was found to be higher during the weekend, compared to weekdays [40]. A national representative survey among Norwegian children found that the intake of sugar-sweetened soft drinks was significantly higher during weekend days, compared to weekdays among four-year old children and school children in 4th and 8th grade [41]. Among 1500 Norwegian adolescents and their parents, the intake of sugar-sweetened beverages was found to be low during weekdays, but doubled during weekend days [42]. In a study among almost 12,000 U.S. adults from 2003–2012 the authors found that energy intake from sugar-sweetened beverages was higher on weekend days compared to weekdays. This difference was larger among men than women [39]. Since the weekend constitutes almost one third of the week, improvement of the composition of foods and beverages consumed during weekends will contribute to improve the total dietary quality. 

### 4.3. Background Variables Associated with the Intake of Different Types of Beverages

Our results implied that water consumption was more prevalent among younger participants and participants having a higher education. This corresponds partly to data from the National Health and Nutrition Examination Surveys (NHANES) from 1999–2006 among more than 4000 U.S. adult participants, where the researchers found that water intake declined with increasing age and higher education was associated with higher water consumption [43]. We also found that women and participants being interested in a healthy diet had higher odds of consuming water.

We observed no associations between milk intake and background variables. Canadian data from 35,000 participants in 2004 described that the proportion of adults who reported drinking milk tended to rise with increasing age. The same study also found that juice consumption was associated with younger age groups [44]. This corresponds to the results from the present study as the odds of juice consumption were significantly lower in the oldest, compared to the youngest, age group.

Coffee intake was associated with higher age, being interested in a healthy diet and smoking in our study. Sousa and Macedo da Costa also found a positive association between coffee intake and higher age among Brazilian adults, but this association was only found for men [45]. A Canadian study of beverage consumption found that coffee consumption peaked at ages 31–50 years and, thereby, decreased with increasing age [44]. Smoking has also been found to be associated with coffee consumption in several other studies [46,47,48].

Tea consumption was associated with all factors analyzed in our study; being a woman, being in the oldest age group, having a normal or low BMI, having higher education, being interested in a healthy diet, and being a non-smoker. Higher tea consumption with increasing age was also reported in the aforementioned Canadian study [44]. A study among almost 6000 university students in Taiwan found that having a higher BMI was a significant predictor of tea drinking [49], which contrasts with our results. De Castro and Taylor describe an association between cigarette smoking and frequent consumption of coffee and tea among 650 U.S. adults [46]. This complies with our results for coffee, but is opposite of our results for tea consumers, where smokers had 54% lower odds of consuming tea, compared to non-smokers.

Consumption of sugar-sweetened beverages dropped sharply at older ages in both the present and other studies [30,44]. Mullie et al. [30] found that high age, high BMI, non-smoking, and income were negatively related to consumption of sugar-sweetened beverages. Our results indicated that participants with university or college education had lower odds of consuming sugar-sweetened beverages. The association between consumption of sugar-sweetened beverages and lower or no education has also been found in other studies [29,31,32]. Liu and coworkers [50] described that, compared to college educated individuals, the odds of consuming sugar-sweetened beverages was more than three times greater for those with high school education or less. Why a lower socioeconomic position is associated with higher consumption of sugar-sweetened beverages is not clear, but it has been argued that the low cost and aggressive marketing in low-income areas could be an explanation [30]. It is well documented that low socioeconomic position is associated with a clustering of unhealthy lifestyles, such as smoking, unhealthy dietary patterns, and obesity [51]. Drinking sugar-sweetened beverages regularly can be seen as an unhealthy habit due to the high energy-content and the low nutritional value [30].

In a study among almost 2000 military men in Belgium, high BMI and trying to lose weight were found to be positively related to consumption of artificially sweetened beverages [30]. This corresponds to our results where participants with a BMI of 25 kg/m^2^ or higher had 70% higher odds of consuming artificially sweetened beverages, compared to participants with a normal or low BMI. This may indicate that people being overweight or obese are drinking more artificially sweetened beverages in an attempt to lose weight [30].

Men in the present study had higher odds of drinking beer compared to women, and the percentage of wine consumers increased with increasing age. The same associations have also been described in the Canadian population [44]. In a study in the older population in Spain from 2008–2010 alcohol consumption was significantly more frequent among men, compared to women [52]. De Castro and Taylor [46] found that cigarette smoking was associated with alcohol consumption among 600 adults in Texas, USA. We found the same tendency for beer consumers, but not for wine consumers.

### 4.4. Strengths and Limitations

The detailed information about types and amounts of beverage intakes and meal types are the major strengths of the Norkost 3 survey, in addition to the relatively large sample size. A limitation of the study is the fairly low participation rate of 37%, which limits the generalizability of the results [53]. The proportion of participants in the Norkost 3 study with a college/university education was higher than in the general population [33]. We may assume that a study population with higher education has healthier beverage consumption habits, compared to the general population with lower education. An association between higher education and healthier diets has been found in several studies [27,28,33]. The consumption of sugar-sweetened beverages may have been underestimated in the present study due to the high percentage of participants with a college/university education. A higher percentage of the participants in Norkost 3 belonged to the highest age interval and a lower percentage to the youngest interval, compared to the general population. The background characteristics of the participants in the Norkost 3 survey, compared to the characteristics of the general population have earlier been described in detail [33]. 

Self-reported surveys collecting the intake of fluids are open to potential bias due to over- or under-reporting of certain fluid types [54]. The use of 24 h recall or food frequency questionnaire (FFQ) has been reported to underestimate fluid intakes by as much as 500 mL/day. The reason for this is that fluids are often consumed outside mealtimes and not perceived as a food [29,55]. The 24 h recall method relies on the participants’ ability and willingness to correctly inform the interviewer about all eating and drinking events that occurred on the preceding day [53]. In the present study pure drinking meals were defined as snacks (pure drinking meals representing about 40% of meals defined as snacks). There is some evidence that snacks are more likely to be underreported than main meals [56], if this is the case in the present study total beverage intake may have been underestimated. Alcohol consumption [57] and soft drink consumption [58] have been described, in particular, as being subject to underreporting. The interviewers in the Norkost 3 survey were thoroughly trained on interview techniques and to remind the participants about forgotten food or drink items, which may have reduced the underreporting of snack events [53].

Body weight was self-reported, which may be a limitation for the validity of the estimated BMI because self-reported weight tends to be underestimated [59,60]. This may have contributed to misclassification of participants as normal weight participants and a reduction in the difference between the two groups [53]. 

### 4.5. Practical Implications

The findings from the present study provide insight into the beverage consumption pattern to different meals among a group of Norwegian adults and in different subgroups of the study population. This insight may be useful when developing and revising dietary recommendations. Holmback et al. has suggested that the inclusion of meal-based recommendations may be an advantage in FBDG [23].

Still, the data in Norkost 3 were collected in 2010–2011, and beverage consumption habits in the population may have changed somewhat during the last 5–6 years.

Age and education seemed to be highly associated with consumption of certain beverage types. Being a young adult (18–34 years) was associated with consumption of sugar-sweetened beverages, artificially sweetened beverages, and water, whereas being older was associated with wine consumption. Having a higher education seemed to be associated with a healthier beverage consumption pattern, including water and less sugar-sweetened beverages. On the other hand, higher education was associated with beer and wine consumption. Knowledge about how beverage consumption is associated with age and education helps us understand habits in subgroups of the population. More knowledge from research on these associations may be used when tailoring interventions regarding consumption of sugar-sweetened and alcoholic beverages in the future.

## 5. Conclusions

Beverage consumption patterns in the Norwegian adult population varied between different meal types. Breakfast was the most important meal type for intake of milk and juice, and dinner for sugar-sweetened beverages and wine, whereas snacks contributed most to intakes of water, coffee, artificially sweetened beverages, and beer. Alcohol consumption was higher on weekend days, compared to weekdays among consumers. Higher education was associated with a healthier beverage consumption pattern, but also more frequent alcohol consumption. Higher age was strongly associated with consumption of coffee, tea and wine, whereas younger age was associated with consumption of water and sugar-sweetened beverages. Knowledge regarding beverage consumption patterns in the population and in subgroups of the population may be considered when revising FBDGs in the future.

## Figures and Tables

**Table 1:** Background characteristics of the study population in Norkost 3 (*n* = 1787).

	Men		Women	
	(*n* = 862)		(*n* = 925)	
	*n*	%	*n*	%
Age group (*n* = 1787)				
18–34 years	199	23	208	22
35–54 years	355	41	461	50
55–70 years	308	36	256	28
BMI (*n* = 1756)				
<25 kg/m^2^	344	40	544	61
≥25 kg/m^2^	517	60	351	39
Education level (*n* = 1784)				
High school, technical school, trade	432	50	414	45
school or lower				
University or college	429	50	509	55
Interest in a healthy diet (*n* = 1786)				
No, low or moderate	447	52	335	36
High or very high	414	48	590	64
Smoking habits (*n* = 1787)				
Non-smokers	686	80	724	78
Smokers	176	20	201	22

**Table 2:** Shows mean daily intake of beverages from each meal.

	Breakfast		Lunch		Dinner		Supper		Snack	
	Mean (g)	95% CI	Mean (g)	95% CI	Mean (g)	95% CI	Mean (g)	95% CI	Mean (g)	95% CI
Water	120 *	112, 129	128 *	120, 136	266 *	255, 278	73 *	66, 80	476	454, 499
Coffee	121 *	113, 128	76 *	70, 81	24 *	20, 27	18 *	14, 21	282	267, 298
Tea men	**36**	29, 42	27 *	22, 32	1 *	NA	11 *	8, 15	33	25, 41
Tea women	57 *	49, 64	53 *	46, 60	7 *	4, 10	36 *	31, 41	85	73, 96
Milk	**125**	118, 132	47 *	43, 52	32 *	29, 35	45 *	41, 50	37 *	33, 42
Fruit juice	**55**	50, 59	21 *	18, 24	7 *	6, 9	8 *	6, 10	15 *	13, 17
Sugar sweetened beverages	3 *	1, 4	12 *	10, 15	**50**	45, 56	11 *	9, 14	41 *	36, 46
Artificially sweetened beverages	5 *	4, 7	12 *	10, 15	38 *	34, 43	12 *	10, 15	44	37, 50
Beer	0 *	NA	1 *	NA	26 *	19, 33	9 *	5, 12	47	36, 58
Wine	0 *	NA	1 *	NA	**22**	19, 25	6 *	4, 7	17 *	14, 20

* *p* ≤ 0.001 for difference in average intake between most important meal (bold numbers) and the other meals, tested with mixed models. *NA*: Not applicable because of zero or small values for average consumption. Mean (grams) and 95% confidence interval (95% CI).

**Table 3:** Proportion of participants consuming sugar-sweetened beverages, artificially sweetened beverages, beer, and wine on one and/or both recall days.

	Sugar-Sweetened Beverages		Artificially Sweetened Beverages		Beer		Wine	
	%	*n*	%	*n*	%	*n*	%	*n*
Women	28.8	260	26.2	242	9.2	85	22.1	204
Men	41.0	353	22.2	191	21.1	182	18.1	156

**Table 4:** Background characteristics associated with users and non-users of different beverage types.

Background Variables	*n* (%)	OR (95% CI)	*n* (%)	OR (95% CI)	*n* (%)	OR (95% CI)	*n* (%)	OR (95% CI)	*n* (%)	OR (95% CI)
	**Water**		**Milk**		**Juice**		**Coffee**		**Tea**	
**Gender**										
Men	809 (94)	1.00	737 (86)	1.00	393 (46)	1.00	720 (84)	1.00	242 (28)	1.00
Women	900 (97)	**1.92 (1.16–3.19)**	764 (83)	0.78 (0.54–1.13)	465 (50)	1.03 (0.84–1.23)	731 (79)	0.69 (0.53–0.90)	493 (53)	**2.76 (2.24–3.41)**
**Age (years)**										
18–34	397 (98)	1.00	352 (87)	1.00	218 (54)	1.00	263 (65)	1.00	141 (35)	1.00
35–54	780 (96)	0.48 (0.23–1.00)	680 (83)	0.81 (0.57–1.15)	409 (50)	0.87 (0.68–1.12)	681 (84)	**2.70 (2.0–3.6)**	356 (44)	**1.41 (1.07–1.84)**
55–70	532 (94)	**0.42 (0.20–0.88)**	469 (83)	0.78 (0.54–1.13)	231 (41)	**0.67 (0.51–0.88)**	507 (90)	**4.9 (3.4–7.1)**	238 (42)	**1.62 (1.2–2.2)**
*p trend*	**0.028**		0.207		**0.003**		**<0.001**		**0.002**	
**BMI**										
<25 kg/m^2^	874 (97)	1.00	760 (84)	1.00	496 (55)	1.00	722 (80)	1.00	424 (47)	1.00
≥25 kg/m^2^	804 (94)	0.71 (0.43–1.17)	718 (84)	0.98 (0.75–1.29)	350 (41)	**0.63 (0.51–0.77)**	707 (83)	0.89 (0.68–1.16)	297 (35)	**0.70 (0.57–0.87)**
**Education**										
No or lower degree	790 (93)	1.00	709 (84)	1.00	323 (38)	1.00	670 (79)	1.00	282 (33)	1.00
University or college	916 (98)	**2.46 (1.47–4.14)**	789 (84)	1.00 (0.77–1.30)	535 (57)	**2.03 (1.67–2.48)**	778 (83)	1.26 (0.98–1.63)	451 (48)	**1.58 (1.28–1.95)**
**Interest in healthy diet**										
No, low or moderate	732 (94)	1.00	649 (83)	1.00	345 (44)	1.00	601 (77)	1.00	254 (33)	1.00
High or very high	976 (97)	**1.81 (1.11–2.96)**	851 (85)	1.21 (0.93–1.58)	513 (51)	1.17 (0.96–1.43)	849 (85)	**1.57 (1.2–2.0)**	481 (48)	**1.46 (1.18–1.80)**
**Smoking habits**										
Non-smokers	1362 (97)	1.00	1186 (84)	1.00	702 (50)	1.00	1129 (80)	1.00	632 (45)	1.00
Smokers	1709 (92)	**0.48 (0.29–0.78)**	315 (84)	0.97 (0.60–1.02)	156 (41)	**0.78 (0.61–0.99)**	322 (85)	**1.7 (1.2–2.4)**	103 (27)	**0.46 (0.36–0.61)**
	**Sugar-sweetened beverages ^1^**			**Artificially sweetened beverages ^2^**		**Beer**		**Wine**		
**Gender**										
Men	353 (41)	1.00		191 (22)	1.00	182 (21)	1.00	156 (18)	1.00	
Women	260 (28)	**0.57 (0.46–0.71)**		242 (26)	**1.38 (1.10–1.75)**	85 (9)	**0.34 (0.25–0.46)**	204 (22)	1.13 (0.88–1.15)	
**Age (years)**										
18–34	221 (54)	1.00		113 (28)	1.00	69 (17)	1.00	41 (10)	1.00	
35–54	260 (32)	**0.43 (0.33–0.56)**		225 (28)	0.95 (0.72–1.25)	120 (15)	0.91 (0.65–1.28)	165 (20)	**2.28 (1.56–3.33)**	
55–70	132 (23)	**0.24 (0.18–0.33)**		95 (17)	**0.47 (0.34–0.65)**	78 (14)	0.81 (0.56–1.17)	154 (27)	**3.78 (2.57–5.57)**	
*p trend*	**<0.001**			**<0.001**		0.255		**<0.001**		
**BMI**										
<25 kg/m^2^	318 (35)	1.00		185 (21)	1.00	140 (16)	1.00	206 (23)	1.00	
≥25 kg/m^2^	287 (34)	0.95 (0.76–1.18)		239 (28)	**1.70 (1.35–2.15)**	123 (14)	0.76 (0.58–1.01)	148 (17)	**0.66 (0.51–0.85)**	
**Education**										
No or lower degree	343 (41)	1.00		223 (26)	1.00	115 (14)	1.00	130 (15)	1.00	
University or college	270 (29)	**0.65 (0.53–0.81)**		209 (22)	0.83 (0.66–1.04)	151 (16)	**1.43 (1.08–1.89)**	230 (25)	**1.67 (1.29–2.15)**	
**Interest in healthy diet**										
No, low or moderate	345 (44)	1.00		211 (27)	1.00	131 (17)	1.00	120 (15)	1.00	
High or very high	267 (27)	**0.56 (0.45–0.69)**		222 (22)	**0.79 (0.63–0.98)**	136 (14)	0.89 (0.67–1.17)	240 (24)	**1.48 (1.14–1.91)**	
**Smoking habits**										
Non-smokers	472 (34)	1.00		328 (23)	1.00	189 (13)	1.00	282 (20)	1.00	
Smokers	141 (37)	1.05 (0.81–1.35)		105 (28)	1.17 (0.90–1.54)	78 (21)	**1.79 (1.31–2.43)**	78 (21)	1.17 (0.87–1.58)	

All analyses are adjusted for all background variables. Bold numbers represents statistical significant values. ^1^ Includes sugar-sweetened soft drinks and squash drinks; ^2^ Includes soft drinks and squash drinks without sugar and/or artificial sweeteners.
